# Early Events in *Populus* Hybrid and *Fagus sylvatica* Leaves Exposed to Ozone

**DOI:** 10.1100/tsw.2010.63

**Published:** 2010-04-01

**Authors:** R. Desotgiu, F. Bussotti, F. Faoro, M. Iriti, G. Agati, R. Marzuoli, G. Gerosa, C. Tani

**Affiliations:** ^1^ Dipartimento di Biotecnologie Agrarie, Sezione di Botanica Applicata e Ambientale, Università di Firenze, Italy; ^2^ Dipartimento di Produzione Vegetale, Università di Milano, Italy; ^3^ Istituto di Fisica Applicata “Nello Carrara” - IFAC Consiglio Nazionale delle Ricerche, Firenze, Italy; ^4^ Dipartimento di Matematica e Fisica Università di Cattolica del Sacro Cuore, Brescia and Fondazione Lombardia per l'Ambiente, Milano, Italy

**Keywords:** Evans blue, chlorophyll a fluorescence, visible symptoms, hydrogen peroxide, multispectral fluorescence microimaging, microspectrofluorometry, open-top chambers, ultrastructure

## Abstract

This paper aims to investigate early responses to ozone in leaves of *Fagus sylvatica* (beech) and *Populus maximowiczii* x *Populus berolinensis* (poplar). The experimental setup consisted of four open-air (OA) plots, four charcoal-filtered (CF) open-top chambers (OTCs), and four nonfiltered (NF) OTCs. Qualitative and quantitative analyses were carried out on nonsymptomatic (CF) and symptomatic (NF and OA) leaves of both species. Qualitative analyses were performed applying microscopic techniques: Evans blue staining for detection of cell viability, CeCl_3_ staining of transmission electron microscope (TEM) samples to detect the accumulation of H_2_O_2_, and multispectral fluorescence microimaging and microspectrofluorometry to investigate the accumulation of fluorescent phenolic compounds in the walls of the damaged cells. Quantitative analyses consisted of the analysis of the chlorophyll a fluorescence transients (fast kinetics). The early responses to ozone were demonstrated by the Evans blue and CeCl_3_ staining techniques that provided evidence of plant responses in both species 1 month before foliar symptoms became visible. The fluorescence transients analysis, too, demonstrated the breakdown of the oxygen evolving system and the inactivation of the end receptors of electrons at a very early stage, both in poplar and in beech. The accumulation of phenolic compounds in the cell walls, on the other hand, was a species-specific response detected in poplar, but not in beech. Evans blue and CeCl_3_ staining, as well as the multispectral fluorescence microimaging and microspectrofluorometry, can be used to support the field diagnosis of ozone injury, whereas the fast kinetics of chlorophyll fluorescence provides evidence of early physiological responses.

## INTRODUCTION

The onset of visible foliar symptoms is considered a response indicator for sensitive plant species exposed to high concentrations of tropospheric ozone[1] and, at least in some tree species, it has also been correlated with ozone foliar fluxes[2,3,4]. Foliar symptoms become visible after a complex series of biochemical and cytological events. These events include the accumulation of hydrogen peroxide ($H_2O_2$) in the apoplast and in the chloroplasts[5], and scattered cell death[6,7,8]. Only after some time, the dead cells aggregated in stipples become evident and visible. Among the biochemical responses, the pattern of phenylpropanoids may have special relevance for early diagnostic purposes. These detoxifying substances can be induced by ozone action[9] and can be detected with fluorescence techniques[10,11,12], thus providing a possible tool for “early warning” diagnosis.

Among the photosynthesis processes, the alterations concerning the reduction of net photosynthesis[13,14,15] were associated with the inactivation of the enzyme ribulose-1,5-biphosphate carboxylase/oxygenase (Rubisco)[16,17,18,19], whereas the alterations of photosystem II (PSII) functionality are more subtle. The most widely used parameter derived from the chlorophyll *a* (Chl *a*) fluorescence analysis, i.e., the quantum yield of primary photochemistry (calculated with the expression $[(F_M-F_0)/F_M]$, i.e., $[F_V/F_M]$[20,21], is very stable and, only when the damage is in an advanced stage, does the lowering of the value of this parameter become evident[22,23]. More information can be obtained by applying methods and concepts related to the fast kinetics[24,25], i.e., the analysis of the polyphasic induction OKJIP curves (transients), and the related parameters[23].

Responses of woody plants to ozone are species specific, and depend on the ecological requirements and structural features of the particular species[26]. We assume that some common characteristic responses indicating the onset of basic physiological processes can be identified in the first phases of ozone stress induction. The purpose is to identify these common behavior traits as early response indicators in tree species showing different ozone sensitivities. Using qualitative methods, we would also be able to confirm the results from the field surveys of ozone injuries, as well as their relative microscopic validation already described in the literature[27].

## MATERIALS AND METHODS

### Experimental Setup

The study was performed within the TOP project (Transboundary Ozone Pollution), using the experimental facility at Curno (C.R.IN.ES., Centre for the Research on Effects of Pollutants on Ecosystems, North Italy, 45°41' N, 9°37' E, elevation 245 m a.s.l.). The experimental setup consisted of eight open-top chambers (OTCs): four with charcoal-filtered (CF) air and four with ambient air (not filtered, NF). Four additional plots were kept in natural conditions, without OTCs (open air, OA). The OA plots and the OTCs were subdivided into two blocks: well-watered (W, kept at field capacity during the whole growing period) and dry (D, plants only watered during periods of severe drought as evidenced by plant wilting.). The results reported here relate only to the well-watered plots. The experiment within the OTCs was conducted from April 1 to September 30 in 2004 and 2005. The CF chambers were supplied with approximately 50% of ambient ozone; the NF chambers, on the other hand, were supplied with about 95% of the ozone in the open air. For details and specifications of the technical equipment, see the studies of Gerosa and colleagues[2,3,4,23]. In 2005, when the sampling was done, the value of the exposure parameter AOT40 (accumulated concentrations above the ozone threshold of 40 ppb[28]) for the period April 1 to August 31 was 24.2 ppm.h in OA conditions (16.3 in NF and 1.2 in CF plots). AOT40 levels in NF chambers at each sampling date were 5.78 ppm.h (May 30), 13.3 (June 27), 18.9 (July 25), and 24.2 (August 30). In each chamber (CF and NF) and in the OA plots, four individuals representing each of the following four species were grown: *Populus maximowiczii* Henry x *Populus berolinensis* Dippel (Oxford clone, called poplar in this paper), *Fraxinus excelsior* L. (ash), *Fagus sylvatica* L. (beech), and *Quercus*

*robur* L. (oak). The plants were placed in the chambers (field grown) in spring 2003 as 1-year-old seedlings (in the case of poplar, they were cuttings). Thus, observations were carried out on 2- and 3-year-old seedlings, except for the poplar plants, grown from cuttings and which displayed such a rapid growth that they were coppiced at the end of winter, in early 2004 and in early 2005. So, a full-sunlight exposure was assured for all plants throughout the experiment.

Within this experiment, we analyzed the early responses (both at the cytological and physiological level) of two tree species with different sensitivities to ozone, as shown by the results of previous studies[2,3,4,23]: the poplar clone, very sensitive, and the beech, moderately sensitive.

## Visible Leaf Symptoms

Visual foliar symptoms were assessed in 2005 on the occasion of every measurement on selected branches. Two plants per species from each well-watered OTC were considered. At the end of May, the selected branches displayed fully extended and mature leaves. Symptom assessment was done with reference to available handbooks and photoguides[1]. The results have been reported in previous studies[2,3,4,23] and are given here as reference for the other analyses. In addition, ultrastructural observations were carried out on a sample of leaves (see following paragraph).

## Cell Viability, Ultrastructural Analysis, and $H_2O_2$ Accumulation

All samplings were carried out on two plants per species from each well-watered plot (i.e., four plants for each experimental condition). Two sun leaves per plant were sampled. All leaves were chosen among those fully extended and mature at the end of May. Sampling was done on May 30, June 27, and July 25, 2005.

Leaf discs (15 mm in diameter) were punched with a cork borer from leaves of the poplar and beech plants, avoiding main veins. Evans blue staining was carried out by boiling leaf discs for 2–3 min in a mixture of phenol, lactic acid, glycerol, and distilled water containing 20 mg $dm^{-3}$ Evans blue (1:1:1:1), prepared immediately before use. Tissues were then clarified overnight in a solution of 2.5 g $cm^{-3}$ chloral hydrate in water[29]. Dead cells stained from dark to light blue, depending on the stage of cell membrane degradation[29], while the undamaged cells appeared unstained. All samples were examined with an Olympus BX50 light microscope (Olympus, Tokyo, Japan), equipped with differential interference contrast (DIC) and epipolarization filters.

Samples (cut leaf pieces) destined for observation by transmission electron microscope (TEM) were processed according to standard procedures. They were prefixed in a phosphate buffer (pH 7.2) containing 2.5% glutaraldehyde + 4% paraformaldehyde. After 20 h at 5°C, samples were rinsed twice (2 × 10 min) in the same buffer, then postfixed (2 h) in 2% osmium tetroxide prepared in the same buffer. Subsequently, samples were dehydrated in an increasing ethanol series (10 min at each stage of the fixation series). Finally, after two 5-min rinses in propylene oxide (100%), the samples were embedded in resin, according to Spurr's procedure[30]. A Reichert Ultracut S (Leica, Heerhrugg, CH) microtome was used to cut ultrathin sections (0.09 µm) with a diamond knife. These sections were stained with uranyl acetate (500 mg in 10 ml of distilled water) and lead citrate (saturated solution). These samples were used for the ultrastructural observations. To localize subcellular accumulation of $H_2O_2$, before applying the standard procedure, samples were incubated under vacuum with 0.05 *M* $CeCl_3$ solution (in 0.05 *M* of MOPS, pH 7.0) for 1 h. During incubation, $Ce_3^+$ ions react with $H_2O_2$, forming electron-dense cerium perhydroxide precipitates[31]. All observations were done with an EM-300 Philips (Amsterdam, NL) microscope at 80 kV.

## Multispectral Fluorescence Microimaging and Microspectrofluorometry

Sampling was done on July 25, with the same sampling design already described for the cell viability and ultrastructural analysis. Cross-sections (50-µm thick) of fresh leaf tissue, cut with a vibratory microtome (Vibratome 1000 Plus, Vibratome, St. Louis, MO) were mounted in phosphate buffer (pH 6.8) with the addition of 1% NaCl (w/v) and observed through the inverted epifluorescence microscope as described earlier[10,32]. Fluorescence spectra excited at 365 nm (from a high-pressure Hg lamp) in the 400–800 nm range were measured by integrating the signal over a spot measuring about 25 µm in diameter (lens ×40) of healthy or injured palisade tissues. Under UV excitation at 365 nm, three fluorescence images were sequentially acquired on narrow (10 nm) bands centered at 470, 546, and 680 nm, selected by interference filters. With blue excitation at 436 nm, only the green-yellow (546 nm) and red (680 nm) bands were measured. Monochrome images were then recombined after band-color assignment in a single multicolor image using the RGB technique provided by Image-Pro Plus v.4.0 software (Media Cybernetics, Silver Spring, MD). The blue, green, and red colors were attributed to the 470-, 546-, and 680-nm fluorescence images, respectively.

## Analysis of the Fluorescence Transient

The chlorophyll fluorescence transients were measured on two plants per chamber per each species and ozone treatment (poplar: four plants NF and four plants CF; beech: four plants NF and four plants CF, in well-watered plots), taking four measurements on each plant on each assessment day (poplar: May 30 and July 27, 2005; beech: May 30 and August 30, 2005), with a direct fluorescence fluorimeter (Handy-PEA, Hansatech Instruments, Pentney, U.K.). Before each measurement, the leaves were dark-adapted for 20 min with leaf clips. The rising transients were induced by a red light (peak at 650 nm), at a maximum intensity of 3000 µmol $m^{-2}$ $sec^{-1}$ on the sample surface, provided by an array of three light-emitting diodes; they were recorded for 1 sec, starting from 20 µsec after the onset of illumination. On a logarithmic time scale, the fluorescence transient from $F_0$ (F at 20 µsec, when all the reaction centers of PSII are open, i.e., when the primary acceptor quinone $Q_A$ is fully oxidized) to $F_P$ (where $F_P = F_M$ under saturating excitation light, when the excitation intensity is high enough to ensure the closure of all reaction centers of PSII, i.e., the full reduction of all reaction centers) had a polyphasic behavior[33,34,35]. The fluorescence values at 20 µsec ($F_0$, step 0), 100 µsec ($F_{100}$), 300 µsec ($F_{300}$), 2 msec (step J), 30 msec (step I), and maximal ($F_M$, step P) were taken into consideration. The first part of the transient (O-J) represents the photochemical phase and gives information about the single turnover events (i.e., the primary reduction of $Q_A$); the final part (I-P) represents the thermal phase and is related to multiple turnover events[25]. The analysis of the transient is called the JIP-test[24,25] and refers to a translation of the original data to biophysical parameters — all referring to time zero (onset of fluorescence induction) — which quantify PSII behavior. In this paper, we have considered the maximum quantum yield of primary photochemistry ($\varphi_{Po} = TR_0/ABS = (F_M\text{-}F_0)/F_M) = F_V/F_M$); the efficiency with which a trapped exciton can move an electron into the electron transport chain further than $Q_A^-$, related to the O-J phase ($ET_0/TR_0 = \Psi_{Eo} = ET_0/TR_0 = 1\text{-}V_J$); and the maximum quantum yield of an electron reaching the acceptor side, related to the I-P phase ($\varphi_{Ro} = RE_0/ABS = 1\text{-}F_I/F_M$)[36,37]. The transients were normalized between $F_0$ and $F_M$ to reveal changes in the kinetics of the transients between these two fluorescence extremes. The so-called ΔV (= $V_{treated} - V_{reference}$) curves were constructed by subtraction of the relative variable fluorescence intensity V values recorded in the NF minus CF plants. The transients were also normalized to the single turnover region and expressed as relative variable fluorescence, with W = f(t); where $W = (F_t\text{-}F_0) = V_t/V_J$. The so-called K band occurred in the fast region (100–300 µsec) of the transients.

### Statistics

Microscopic techniques provide qualitative data, indicating the presence or absence of a certain response. Quantitative data are provided only for the chlorophyll *a* fluorescence analysis. Significance of the differences was assessed with a one-way ANOVA, Tukey-test, HSD (Honest Significant Difference), using the software Statistica 7.1 (StatSoft, Tulsa, OK).

## RESULTS

### Visible Leaf Symptoms and Ultrastructural Features

In poplar, symptoms consisted of interveinal browning appearing in late June and affecting the adaxial surface, later spreading and degenerating into necrotic patches. Symptoms appeared early and increased progressively over the entire season in the ozone-exposed plots, affecting primarily the leaves in the lower canopy, which began to shed as early as mid-July. In beech, visible foliar symptoms only appeared in the late part of the growing season (late July) and consisted of fine dark stippling and, more frequently, of browning on the adaxial surface of the leaf; they affected the entire leaf lamina, mostly in the upper part of the crown. No cases of early abscission were observed. Microscopic analysis revealed broad collapsed areas in the palisade mesophyll of poplar leaves, which also involved the inner cells of the spongy tissue. The damaged cells were separated from the healthy ones by means of a callose layer. In the leaves of beech that had turned brown, the most important changes were observed in the palisade cell areas in contact with the upper epidermis. The cells were still intact, but the cytoplasmatic contents appeared degenerated. The vacuoles were enlarged toward the areas in contact with the upper epidermis, and a callose layer was observed between the vacuole and the cytoplasm. Palisade cell death and thickening of the cell walls were detected only in leaves displaying the “stipple” symptomatology. Micrographs of these features have already been reported, for these and other species[13,27,32].

### Cell Viability and $H_2O_2$ Accumulation

Poplar leaf tissues collected on May 30 from CF plots appeared completely undamaged (Fig. 1A), while those from NF plots already showed light blue staining of numerous palisade mesophyll cells (Fig. 1B), indicating an altered membrane permeability. In samples collected from OA plots, some of these cells were already dead, since they stained dark blue (Fig.1C); although their number was not high enough for the appearance of visible symptoms. Symptoms became clearly visible in the following weeks, thus no more observations investigating cell death were carried out on this species.

Beech samples collected on May 30 from all the different plots did not reveal any damaged or dead cells (not shown). Instead, for those collected on June 27 from NF plots, some palisade mesophyll cells appeared damaged (Fig. 1F) and a few of them were dead. At the same sampling time, leaf tissues from OA plots already revealed small groups of dead cells intermingled with numerous others showing altered membrane permeability (Fig. 1H), whereas no dead cells were detected in the samples from CF plots (Fig. 1D). No visible symptoms were apparent at this date in samples from any plot. In samples collected from NF plots a month later (July 25), and still symptomless, numerous clusters of dead cells were detected (Fig. 1G). Some of these cells stained red, possibly due to the reaction of the staining with the polyphenolic compounds accumulated inside. It is worth noting that these leaves manifested stipple symptoms a week later. Large reddish clusters of mesophyll cells were the main traits of tissue samples from OA plots (Fig. 1I). These aggregates were responsible for the typical stipples already visible in the leaves. No or few responses were observed in the CF plots (Fig. 1E).

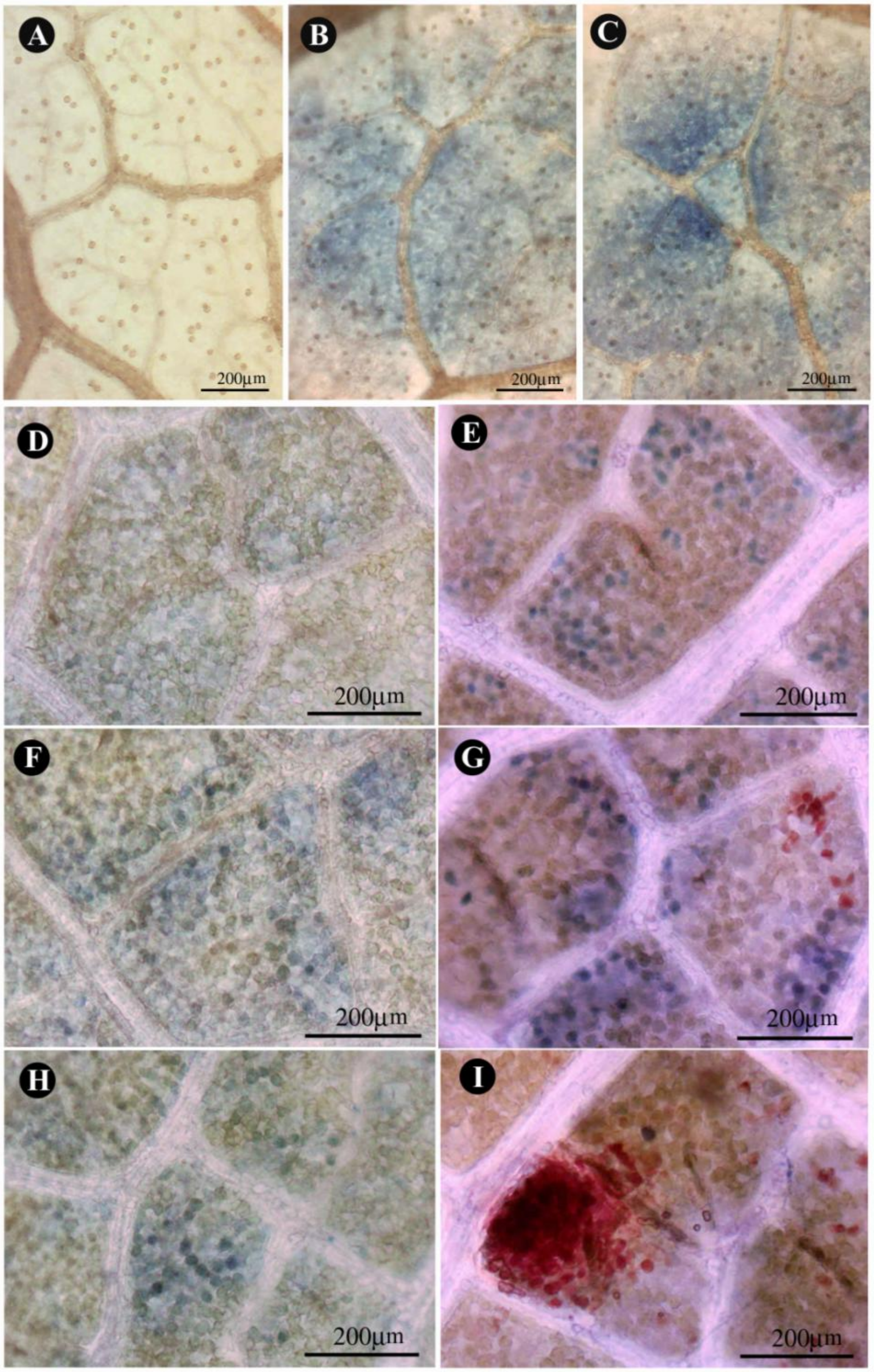

**FIGURE 1.** Cell viability analysis on leaf tissues. (A–C) Samples of poplar collected on May 30, before the onset of visible symptoms, from CF (A), NF (B), and OA plots (C). Dead cells, stained in dark blue by Evans blue staining, are present only in the OA sample, while those slightly damaged (light blue) are numerous also in the NF tissues. No cell damages are visible in the CF sample. (D–I) Samples of beech collected on June 27, before the onset of visible symptoms (D, F, H), and on July 25, after the onset of visible symptoms (E, G, I), from CF (D, E), NF (F, G), and OA plots (H, I). Dead cells, stained in dark blue, are rare on the first sampling date and present only in NF and OA plots. A month later, some dead cells are visible also in the CF sample (E), while they are very numerous in leaf tissues from the NF plot (G), together with small groups of red cells that will possibly generate visible stipples, as those shown in (I) from the OA plot from the same date.

Fig. 2 shows the evolution of $H_2O_2$ in leaves of the two species exposed to ozone, observed by TEM and with $CeCl_3$ staining[31]. In both species, $H_2O_2$ was always absent in samples collected from CF chambers (Figs. 2A, poplar, and 2E, beech). The behavior of the two species was analogous in NF chambers and in OA plots. In poplar and beech, $H_2O_2$ stained positively to $CeCl_3$ already in the May sample (Figs. 2B, poplar, and 2F–H, beech, arrows). Electron-dense deposits were visible on the outer part of the cell walls, especially in the spongy parenchyma and near vessel bundles. In samples collected later in the season (July 27), the deposits of $CeCl_3$ were much more extensive in poplar and penetrated in the inner side of the cell wall adjacent to the plasma membrane (Fig. 2C, D, arrows), while they were not clearly recognized in beech (not shown). In this latter species, there was an electron-dense response observable even in samples not treated with $CeCl_3$, thus suggesting that there was a combination of different responses.

## Multispectral Fluorescence Microimaging and Microspectrofluorometry

No changes were observed in asymptomatic leaves. Fig. 3 shows the UV-induced autofluorescence microscopic analysis of ozone-injured leaves from beech (Fig. 3A–D) and poplar (Fig. 3E–G). Under the assumption that the fluorescence signal is proportional to the chlorophyll content[38], in all the investigated samples, the UV-excited autofluorescence spectra indicated a considerable (about seven times) reduction of chlorophyll (Chl) inside the injured palisade tissue as compared to healthy tissue. Multispectral fluorescence imaging and the related intensity profiles also showed the almost complete disappearance of chlorophyll in the damaged palisade, while intact chloroplasts were present in the palisade cells next to the necrotic ones and in the spongy parenchyma even beneath the injured palisade. In poplar, an intense blue fluorescence (around 450 nm) was observed in the healthy cells of the first palisade layer (Fig. 3E–G). This fluorescence was localized in the cell vacuole, as illustrated by the enlargement shown in Fig. 3G. The same blue fluorescence was observed in the healthy zones of beech leaves. The blue emission is largely reduced in the injured palisade areas of beech, while green-yellow emission was relatively less affected (see Fig. 3E and insets of Fig. 3A, C). Fig. 3F shows that the green-yellow autofluorescence was markedly enhanced in the damaged palisade of poplar leaves and that this emission appeared localized on the cell walls rather than in the cell vacuole. No similar changes in green-yellow fluorescence between necrotic and healthy zones were found for beech.

The different response to ozone stress between poplar and beech in accumulating fluorescence compounds is also highlighted in Fig. 4A, B, where green and red fluorescence images under blue excitation (436 nm) are superimposed. Again, the increase of green-yellow fluorescence was observed in the injured palisade of poplar, but not in that of beech. It is worth noting that the necrotic area of poplar leaf sections appeared orange-brownish under transmitted light microscopy, and that the pigment concentration was higher at the interface between the first and the second palisade layer, as shown by the dark area in the image of Fig. 4C. We also found that the dark-pigmented zone colocalizes with the highest green-yellow fluorescence area (Fig. 4D).

## Significant Differences of Chlorophyll *a* Fluorescence Parameters

The analysis of the fluorescence transients was performed before the onset of visible symptoms (May 30), and when the symptomatic expression was widespread and clear (July 24 on poplar and August 30 on beech). The shape of the mean original transient is shown in Fig. 5A, B, and the JIP-test parameters are reported in Table 1. $F_M$ declined significantly in the ozone-treated poplar trees in July, whereas $F_0$ was higher at the beginning of the season in beech in the NF chambers. The maximum quantum yield of primary photochemistry ($F_V/F_M$ or $TR_0/ABS$) was a very stable parameter and declined only in the ozone-treated poplar plants at the end of the season, with a behavior close to $F_M$. Early in the season, $ET_0/TR$ had decreased significantly, but only in the ozone-treated poplar. $RE_0/ABS$, however, declined in all species in NF conditions.

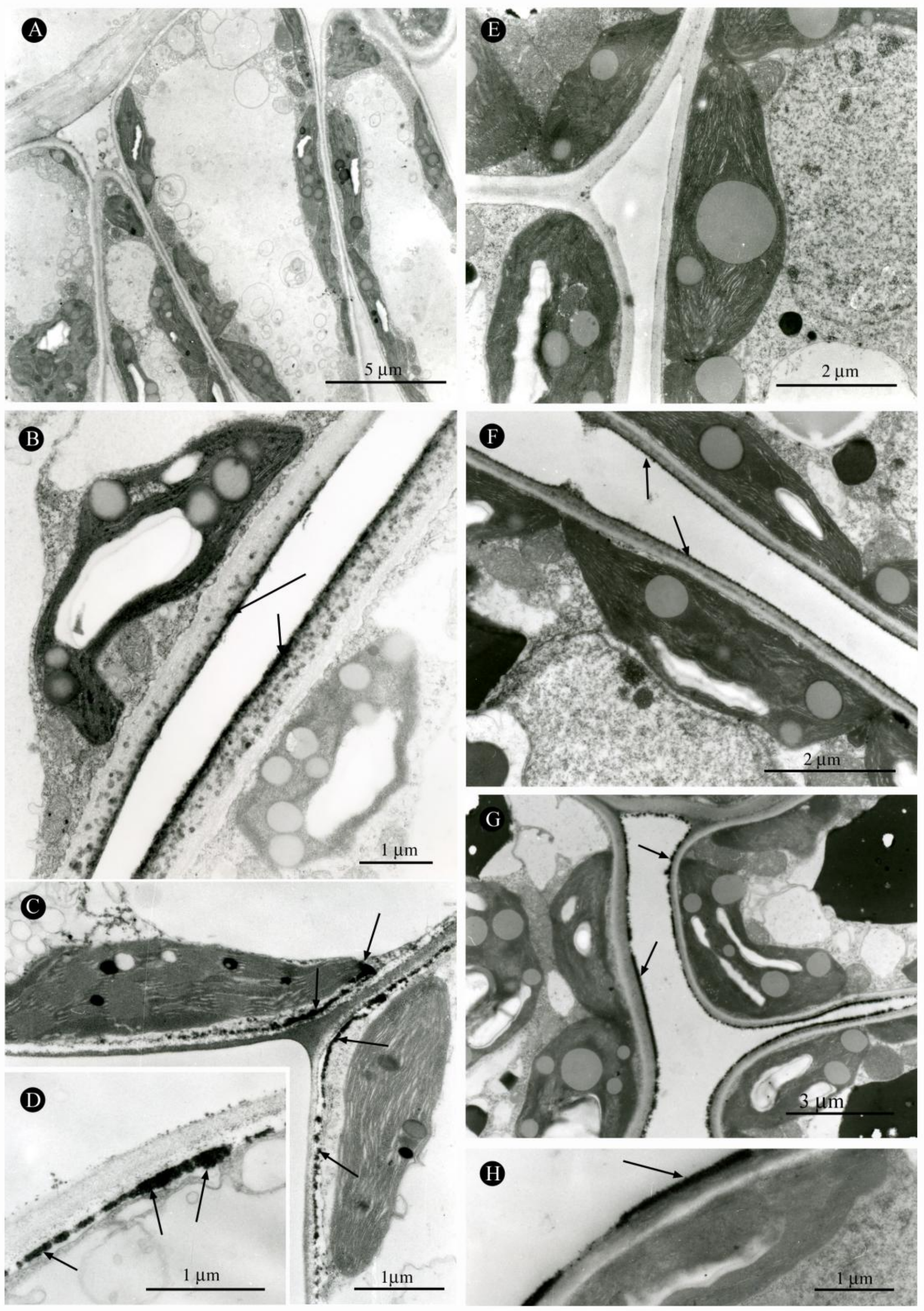

**FIGURE 2.** TEM of poplar (A–D) and beech (E–H) leaves, stained with $CeCl_3$ to evidence the presence and localization of $H_2O_2$. (A, E) CF chambers (control), (B–D, F–H) NF and OA chambers. (B, F–H) Samples collected May 30 (before the onset of visible symptoms). (C, D) Samples collected July 27. The dark deposits on the cell walls (arrows) indicate the presence of $CeCl_3$ oxidized with $H_2O_2$.

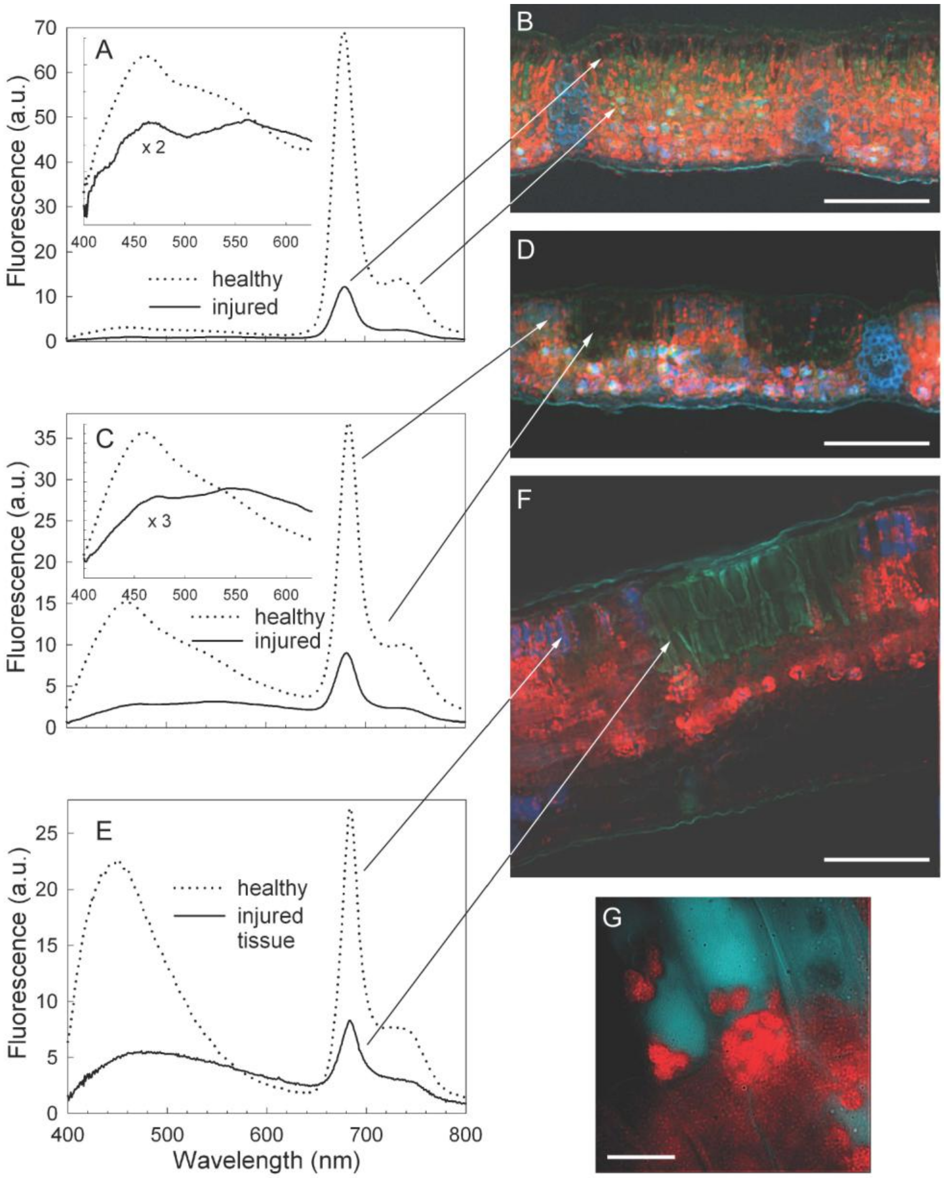

**FIGURE 3.** Multispectral autofluorescence microimaging and microspectrofluorometry of beech (A–D) and poplar (E–G) leaves injured by ozone. (A, B) Cross-section from a beech leaf with bronzing symptoms. (C–G) Cross-section from a beech (C, D) and a poplar (E–G) leaf with stippling symptoms. (A, C, E) UV-induced (365 nm) autofluorescence spectra recorded in the palisade parenchyma from healthy (dotted curves) and injured (solid curves) tissues; insets show the spectral shape in the blue-yellow range with the injured tissue fluorescence enhanced by a factor of 2 and 3 for beech with bronzing and stippling symptoms, respectively. (B, D, F) Fluorescence images obtained by RGB recombination of UV-excited (365 nm) monochrome images recorded at 470, 546, and 680 nm colored in blue, green, and red, respectively; bars = 100 µm. (G) Fluorescence image of the healthy palisade in poplar obtained by RB recombination of UV-excited monochrome images recorded at 470 and 680 nm colored in blue and red, respectively; bar = 10 µm.

The $\Delta V$ curves (Fig. 5C, D) indicate clear $\Delta V_I$ peaks of fluorescence, both in poplar and beech, whereas the $\Delta V_J$ peak was visible only in poplar. In the $\Delta W$ curves (Fig. 5E, F), the $\Delta W_K$ peak, at 0.2–0.4 msec, representing the inactivation of the oxygen evolving system (OES)[25,39], was evident in both species already at the first assessment date. All the peaks described here increased over time and were more pronounced in poplar than in beech.

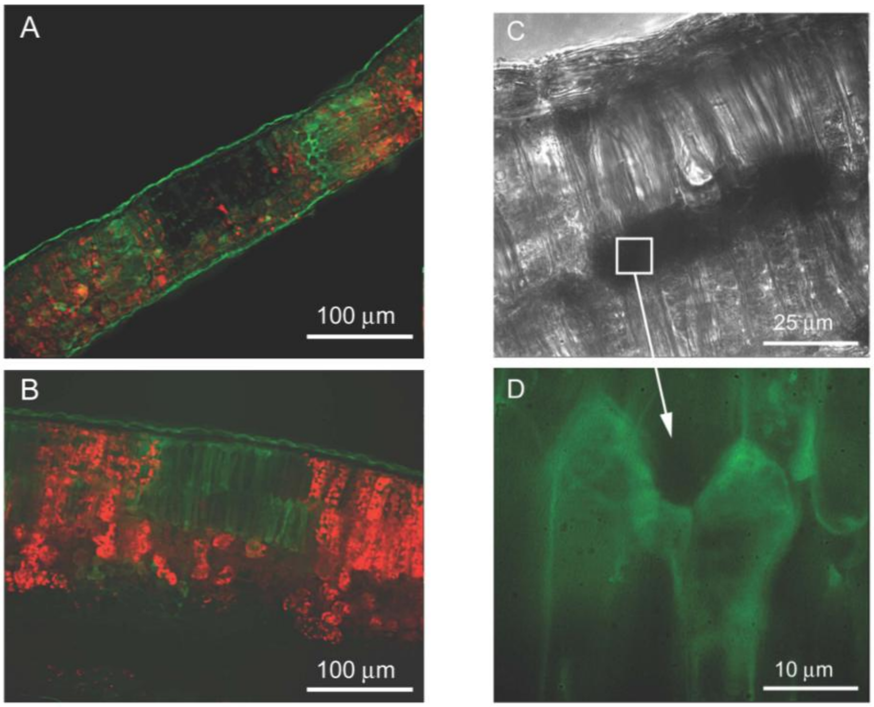

**FIGURE 4.** Autofluorescence images obtained by merging two bands of blue-excited (436 nm) monochrome images recorded at 546 and 680 nm, colored in green and red, respectively, for a beech (A) and a poplar (B) leaf cross-section. (C) Transmitted light image recorded at 546 nm from an injured area of a poplar leaf cross-section. (D) Green-yellow autofluorescence at the interface between the first and second palisade layers, as indicated in (C) (square).

## DISCUSSION

Several reactions precede the onset of foliar symptoms. In both species, symptoms were preceded by cell content alterations highlighted a month before by means of Evans blue. By TEM observations, electron-dense $CeCl_3$ deposits were found in both species in the outer portions of the mesophyll cell walls, revealing an apoplastic production of $H_2O_2$[31].

The apoplast is a site of environmental sensing[40], and $H_2O_2$ is an important signaling molecule for various biotic and abiotic stresses[41,42,43,44,45,46]. $H_2O_2$ itself is only slightly toxic if kept at low concentrations, while excess $H_2O_2$ accumulation, as a reactive oxygen species (ROS), can lead to oxidative stress in plants, triggering cell death[47]. The $CeCl_3$ deposits observed in our samples can be due to the inhibition of $H_2O_2$ utilization by antioxidant enzymes, or to an enhancement of $H_2O_2$ production via an enzymatic or a chemical mechanism[44]. Under severe stress conditions, both processes can be present. In our study, the localization of $CeCl_3$ deposits was more intense in the vascular tissue cell walls and in the cells nearest to the spongy mesophyll. Formation of $H_2O_2$ can occur even up to a certain distance from the cell affected by the stimulus, even in the absence of a direct symplastic connection[48], often in the apoplast of cells near the veins[49] and in the spongy parenchyma[50]. In severe oxidative conditions, $CeCl_3$ deposits accumulate on cell walls and penetrate into the inner side of the cell wall, adjacent to the plasma membrane with a pattern described by several authors. The cytochemical localization of $H_2O_2$ by $CeCl_3$ staining revealed that in ozone-sensitive and ozone-tolerant clones of poplar, extracellular $H_2O_2$ accumulation was one of the earliest detectable responses to ozone; but in the ozone-tolerant clone, $CeCl_3$ precipitates were fewer in number and smaller[51]. Similar evidence was reported for sunflower (*Helianthus annuus*)[52]. In ozone-sensitive clones of *P. tremuloides*, the $CeCl_3$ deposits penetrate into the innermost layers of the cell wall, even reaching the plasma side of the wall, while

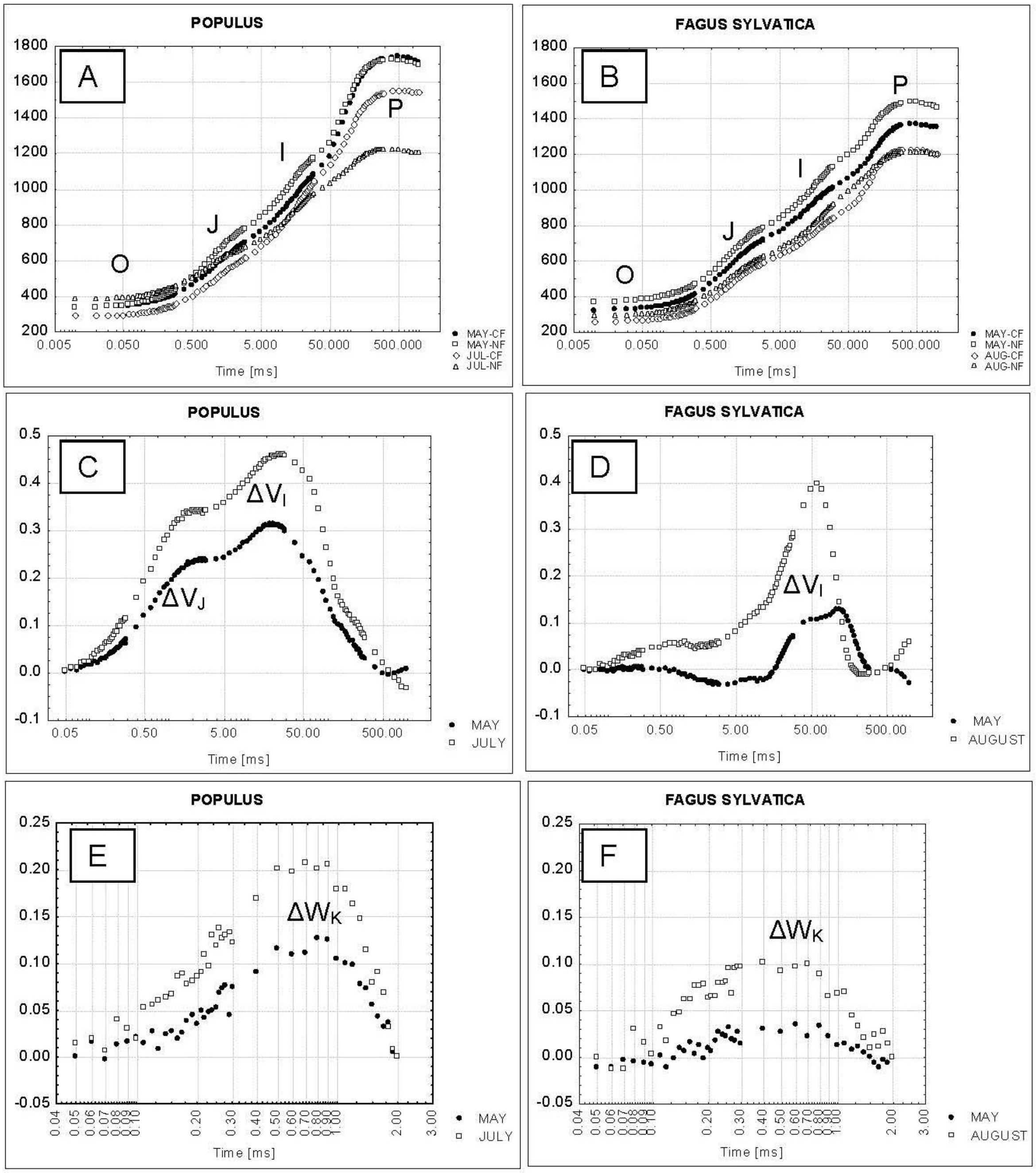

**FIGURE 5**. (A, B) Original transient mean curves, per month and experimental conditions (CF and NF). The O-J-I-P steps are labeled. (C, D) ΔV curves, obtained by subtraction from the original fluorescence transients (NF-CF) normalized between $F_0$ and $F_M$ and multiplied by 4. (E, F) ΔW curves, obtained by subtraction from the original fluorescence transients (NF-CF) normalized between $F_0$ and $F_J$ and multiplied by 4. Assessment dates: May 30, before the onset of symptoms; July 24 (poplar) and August 30 (beech), when symptoms were fully expanded.

in ozone-resistant clones, the deposits remain in the outermost portions of the wall[53]. Unlike poplar, the evolution of $H_2O_2$ over time was not clear in beech, since in the latest samples, an intense electron-dense reaction was detected even in the absence of $CeCl_3$. Our findings may indicate the presence of substantial enzyme activity in the walls, producing an array of molecules differentially involved in ozone tolerance (phenolics, lignin, pathogenesis-related proteins, phytoalexins), many of which are strongly osmiophilic.

**TABLE 1**
**JIP-Test Parameters Calculated from the Original Transients (Mean and Standard Error, n = 4)**

| | | | $F_M$ | | $F_0$ | | $TR_0/ABS$ | | $ET_0/TR_0$ | | $RE_0/ABS$ | |
|---|---|---|---|---|---|---|---|---|---|---|---|---|
| | | | M | ± SE | M | ± SE | M | ± SE | M | ± SE | M | ± SE |
| Beech | May | CF | 1368 | ± 21 | 330.44 | ± 5.97 | 0.76 | ± 0.001 | 0.67 | ± 0.021 | 0.26 | ± 0.020 |
| | | NF | 1489 | ± 90 | 380.5 | ± 19.04 | 0.74 | ± 0.011 | 0.67 | ± 0.023 | 0.24 | ± 0.019 |
| | | | | | ** | | | | | | *** | |
| | August | CF | 1227 | ± 36 | 267.13 | ± 5.81 | 0.78 | ± 0.007 | 0.7 | ± 0.012 | 0.31 | ± 0.014 |
| | | NF | 1217 | ± 62 | 309.92 | ± 10.83 | 0.74 | ± 0.011 | 0.67 | ± 0.012 | 0.23 | ± 0.023 |
| | | | | | * | | | | | | ** | |
| Poplar | May | CF | 1738 | ± 32 | 341.29 | ± 17.25 | 0.8 | ± 0.013 | 0.78 | ± 0.006 | 0.38 | ± 0.012 |
| | | NF | 1655 | ± 63 | 342.69 | ± 8.73 | 0.79 | ± 0.004 | 0.72 | ± 0.022 | 0.33 | ± 0.008 |
| | | | | | | | | | * | | * | |
| | July | CF | 1565 | ± 26 | 294.5 | ± 5.05 | 0.81 | ± 0.003 | 0.78 | ± 0.009 | 0.33 | ± 0.009 |
| | | NF | 1205 | ± 50 | 264.46 | ± 13.61 | 0.77 | ± 0.018 | 0.69 | ± 0.024 | 0.23 | ± 0.029 |
| | | | *** | | | | ** | | ** | | ** | |

*Note:* $TR_0/ABS = \varphi_{Po}$ = maximum quantum yield of primary photochemistry; $ET_0/TR_0 = \Psi_{Eo}$ = efficiency with which a trapped exciton can move an electron into the electron transport chain further than $Q_A^-$; $RE_0/ABS = \varphi_{Ro}$ = maximum quantum yield of an electron reaching the acceptor side. Asterisks (*) indicate the significance of differences between CF and NF treatment (Tukey test, HSD). * = $p < 0.05$; ** = $p < 0.01$; *** = $p < 0.001$. Measurement dates were May 30 and August 30, 2005 for beech; May 30 and July 25, 2005 for poplar. Data expressed as a.u. (arbitrary units).

The analysis of the fluorescence transients demonstrated that, whereas the maximum quantum yield of primary photochemistry ($F_V/F_M$) was a very stable parameter and significant changes occurred only when leaf damage was evident[23], changes in the shape of the I-P region were early responses to ozone. This behavior is expressed by the lower value of the maximum quantum yield of an electron reaching the acceptor side beyond the PSI - $RE_0/ABS$, and by the marked negative peak $\Delta V_I$ in the latest part of the $\Delta V$ curve (NF-CF). A $\Delta V_I$ peak already became evident in the May assessment in both species. The I-P region of the fluorescence transient reflects the velocity of the reduction of ferredoxin beyond PSI[54,55]; in other words, it expresses a relative abundance of PSI relative to PSII. In our case, this behavior can also be connected to a reduced request of electrons to feed the Calvin cycle, since the enzyme Rubisco is inactivated by ozone stress[17,18,19,56,57]. If the flow of electrons through the electron transport chain exceeds the capacity of the metabolism to consume the reductant produced, potentially harmful side reactions are liable to occur[58]. This behavior can also explain the decline of $P_N$, also considered a response to ozone that in some studies is reported to occur early in the growing season[13,15,23]. In a previous study, $RE_0/ABS$ was the parameter that correlated most closely with $P_N$[59].

In the single turnover region (relative variable fluorescence normalized between $F_0$ and $F_J$), $\Delta W$ curves showed the early onset of a pronounced K band in the ozone-treated plants. This indicates a decrease of energetic cooperativity (i.e., the connection among the reaction center, the core antenna complex, and light harvesting compounds[39]), and the disconnection of the reaction centers from the core antenna complexes. After the initial fast fluorescence rise, which is due to the accumulation of reduced $Q_A$, the fluorescence intensity decreases, forming a band that is identified as K at a very early stage.

The multispectral fluorescence microimaging and microspectrofluorometry analyses revealed similarities and differences between the two species. Obviously, the most relevant common trait consists in the reduction of chlorophyll inside the injured zone as compared to the healthy one. The differences are related to the phenolics pattern. In beech, the decrease of the blue autofluorescence of the necrotic cells may be due to the loss of soluble polyphenols (hydroxycinnamates) and/or to a modification of the cell walls. The relatively higher intensity of green-yellow fluorescence, as compared to the blue one, in the injured tissues can also indicate a change in the wall-bound phenolic compounds induced by the ozone stress. In poplar, the accumulation of autofluorescent compounds in the palisade cells beneath the stipples is similar to that recently observed in *Acer pseudoplatanus* leaves affected by ozone[26]. It is widely reported that an increase in fluorescing compounds represents a sign of the hypersensitive response and oxidative burst resulting from pathogen infection[60], as well as a response to ozone stress[61]. A conclusive identification of these substances has not been reported yet, but they appear to have a phenolic nature[62]. Hydroxycinnamic acids released from the vacuoles and esterified on the cell walls were associated to the blue light–excited yellow autofluorescence in lettuce undergoing hypersensitive responses[60]. This indication would fit well with our observation of the decrease in the vacuolar blue autofluorescence, due to hydroxycinnamates, within the ozone-injured palisade cells. Nevertheless, to explain the shift in fluorescence color from blue to green-yellow, we must assume that the binding of hydroxycinnamic acids to the cell walls changes their fluorescence properties as compared to those in solution (cell vacuole or *in vitro*)[10,63]. Alternatively, the occurrence of the green-yellow autofluorescence in the necrotic palisade tissue of poplar can be due to other phenolic compounds, such as flavonoids, which do not show any appreciable emission in a free state (cell vacuole), but fluoresce in a more rigid environment; for example, when bound to cell structures or in an aggregated form. This hypothesis is supported by the recent observation of an orange autofluorescence of flavonoid compounds in the trichome arms of *Cistus salvifolius* leaves[64]. Yet here, the greatest green-yellow fluorescence was found in the palisade areas with the highest pigment concentration, where aggregation is more likely.

## CONCLUSIONS

Poplar and beech have different sensitivities to ozone[65], but their responses have several common traits, although of different magnitude. The loss of cell viability demonstrated with Evans blue, and the formation and accumulation of $H_2O_2$ in the apoplastic compartment, were typical common responses at the cellular level, and the presence of a $\Delta V_I$ peak and a $\Delta W_K$ band were characteristic of early physiological responses. Less promising as a diagnostic tool is the behavior of the fluorescence of secondary metabolites. Responses were obtained only at high damage rates and only on poplar.

Our results demonstrated that the cell viability analysis (with Evans blue staining) and $H_2O_2$ accumulation (with $CeCl_3$ staining) are reliable qualitative tests. These tests enable us to reveal the presence or absence of certain responses, so supporting the traditional approaches, based on visible symptom assessment and subsequent microscopic validation[27].

## ACKNOWLEDGMENTS

The OTC facilities at Curno, where this work was carried out, are funded by Regione Lombardia within the programme "TOP”, in collaboration with the Regional Agency for Environment Protection (A.R.P.A.), the Lombardy Environment Foundation (F.L.A.), and the Regional Agency for Services in Agriculture and Forests – E.R.S.A.F. The authors are grateful to E.R.S.A.F. personnel for their valuable assistance at the Curno nursery.
